# Lupus podocytopathy superimposed on diabetic glomerulosclerosis: A case report

**DOI:** 10.1097/MD.0000000000027077

**Published:** 2021-09-17

**Authors:** Lin Liu, Brian Murray, John E. Tomaszewski

**Affiliations:** a Department of Pathology and Anatomical Sciences, Jacobs School of Medicine and Biomedical Sciences, University at Buffalo, State University of New York, Buffalo, NY; b Department of Internal Medicine, Jacobs School of Medicine, University at Buffalo, State University of New York, Buffalo, NY.

**Keywords:** case report, coexistent glomerular diseases, differential diagnoses, lupus podocytopathy

## Abstract

**Rationale:**

Lupus podocytopathy (LP) is an entity that is increasingly being reported in the literature on systemic lupus erythematosus (SLE). LP is characterized by nephrotic syndrome in SLE patients with diffuse glomerular podocyte foot process effacement and no immune complex deposits along the capillary loops. Histologically, LP typically mimics minimal change disease or primary focal segmental glomerulosclerosis (FSGS) on a background of ISN/RPS class I or II lupus nephritis. In situations where there are coexistent glomerular diseases, however, LP may be easily masked by background lesions and overlapping clinical symptoms.

**Patient concerns:**

We report the case of a 24-year-old woman with type I diabetes, hypertension, psoriasis/rash, and intermittent arthritis who presented with abrupt onset of severe nephrotic proteinuria and renal insufficiency. Renal biopsy revealed nodular glomerulosclerosis and FSGS. Immune deposits were not identified by immunofluorescence or electron microscopy. Ultrastructurally, there was diffuse glomerular basement membrane thickening and over 90% podocyte foot process effacement. With no prior established diagnosis of SLE, the patient was initially diagnosed with diabetic nephropathy with coexistent FSGS, and the patient was started on angiotensin-converting enzyme inhibitors (ACEI) and diuretics. However, nephrotic proteinuria persisted and renal function deteriorated. The patient concurrently developed hemolytic anemia with pancytopenia.

**Diagnoses:**

Subsequent to the biopsy, serologic results showed positive autoantibodies against double strand DNA (dsDNA), Smith antigen, ribonucleoprotein (RNP), and Histone. A renal biopsy was repeated, revealing essentially similar findings to those of the previous biopsy. Integrating serology and clinical presentation, SLE was favored. The pathology findings were re-evaluated and considered to be most consistent with LP and coexistent diabetic nephropathy, with superimposed FSGS either as a component of LP or as a lesion secondary to diabetes or hypertension.

**Interventions:**

The patient was started on high-dose prednisone at 60 mg/day, with subsequent addition of mycophenolate mofetil and ACEI, while prednisone was gradually tapered.

**Outcomes:**

The patient's proteinuria, serum creatinine, complete blood counts, skin rash, and arthritis were all significantly improved.

**Conclusion:**

The diagnosis of LP when confounded by other glomerular diseases that may cause nephrotic syndrome can be challenging. Sufficient awareness of this condition is necessary for the appropriate diagnosis and treatment.

## 1 Introduction

Lupus podocytopathy (LP) is a newly proposed entity in the lupus nephritis spectrum not yet included in the updated 2018 edition of the ISN/RPS classification of lupus nephritis. It represents approximately 1% of renal biopsies from SLE patients.^[1,2]^ LP encompasses a unique presentation of lupus nephritis characterized by nephrotic syndrome with at least 50% podocyte foot process effacement^[2]^ (or over 70% by Columbia University criteria^[1]^) and immune complex deposition limited only to the mesangium. In rare cases, LP can be diagnosed without immune complex deposition. The category of LP confounds the canonical view that massive proteinuria in SLE is related to capillary loop involvement with immune complex deposition. The histopathology of LP may resemble minimal change disease, primary focal segmental glomerulosclerosis, or mesangial proliferative glomerulopathy. In practice, however, SLE patients may have additional systemic diseases that exert superimposed damage on the kidneys, resulting in a more complicated glomerular pathology and obscuring a diagnosis of LP. In this report, we present a case of LP that was diagnosed in a patient with long-term diabetes, hypertension, and an undefined connective tissue disease that subsequently emerged as SLE.

## 2 Case presentation

### 2.1 Initial presentation

A 24 year old Caucasian woman with a medical history of type 1 diabetes for 14 years on insulin pump, hypertension, Raynaud's syndrome, arthritis mainly affecting the proximal interphalangeal joints of both hands, and skin rash along the hairline in the occipital area thought to represent psoriasis, presented to the emergency department with sudden onset of bilateral lower extremity swelling and associated abdominal cramping pain for 1 week. Two weeks prior, adalimumab prescribed for arthritis/skin rash had been discontinued, and the patient was started on prednisone 15 mg daily. The patient dated the lower extremity swelling to shortly after the initiation of prednisone. She denied a history of kidney problems or a family history of kidney disease. On physical examination, her blood pressure was 142/93 mm Hg. There was bilateral lower extremity 2+ pitting edema and bilateral hand swelling. There were no other findings on physical examination. Her lab data showed renal insufficiency with serum creatinine of 1.9 mg/dL (baseline was 0.9 mg/dL) and her eGFR was calculated to be 32.5 mL/min/1.73 m^2^. There was evidence of nephrotic syndrome with a serum albumin level of 1.8 g/dL, urine protein level of 3+ (773 mg/dL), urine albumin/creatinine ratio of 4296 mg/g Cr, and hyperlipidemia. Urine analysis showed hyaline casts 2–5/lpf, urine blood 2+, RBC about 2–5/hpf, WBC >50 hpf, leukocyte esterase 1+, and urine bacteria Few/hpf, but no red cell casts. Laboratory tests also revealed poorly controlled diabetes with a bedside glucose level of 230 mg/dL, HbA1c level of 9%, and urine glucose level of 338 mg/dL. There was mild normocytic normochromic anemia with an RBC count of 3.08 × 10e12/L and hemoglobin level of 9.3 g/dL. Platelet count was normal, and there was mild neutrophilia of 11.6 × 10e9/L and 91.2%. Laboratory tests 1 year previously were reportedly positive for ANA, SSA, anti- double strand DNA (dsDNA), and ribonucleoprotein (RNP), although with normal complements and renal function at the time. There was, however, no clinically established diagnosis of systemic lupus erythematosus (SLE) at the time of the first renal biopsy. Samples for repeat serology tests were collected the day before the biopsy. The clinical differentials at the time of the biopsy included diabetic nephropathy, minimal change disease, membranous nephropathy, focal segmental glomerulosclerosis, and lupus nephritis, including the possibility of prior TNF (adalimumab) therapy-induced lupus-like nephritis. A renal biopsy was performed. Several serology test results became available at the time when the biopsy was evaluated, and of these, ASO titers, ANCAs, anti-GBM, cryoglobulins, and rheumatoid factor were all negative; C3 and C4 were within normal limits; the ANA titer, however, was strongly positive at 1:20480. Other autoantibodies, including anti-dsDNA, anti-histone, Jo-1, SSA/SSB, anti-Smith, anti-RNP, and anti-PLA2R, were still pending at the time of discharge.

### 2.2 Pathological findings

**Figure 1:**
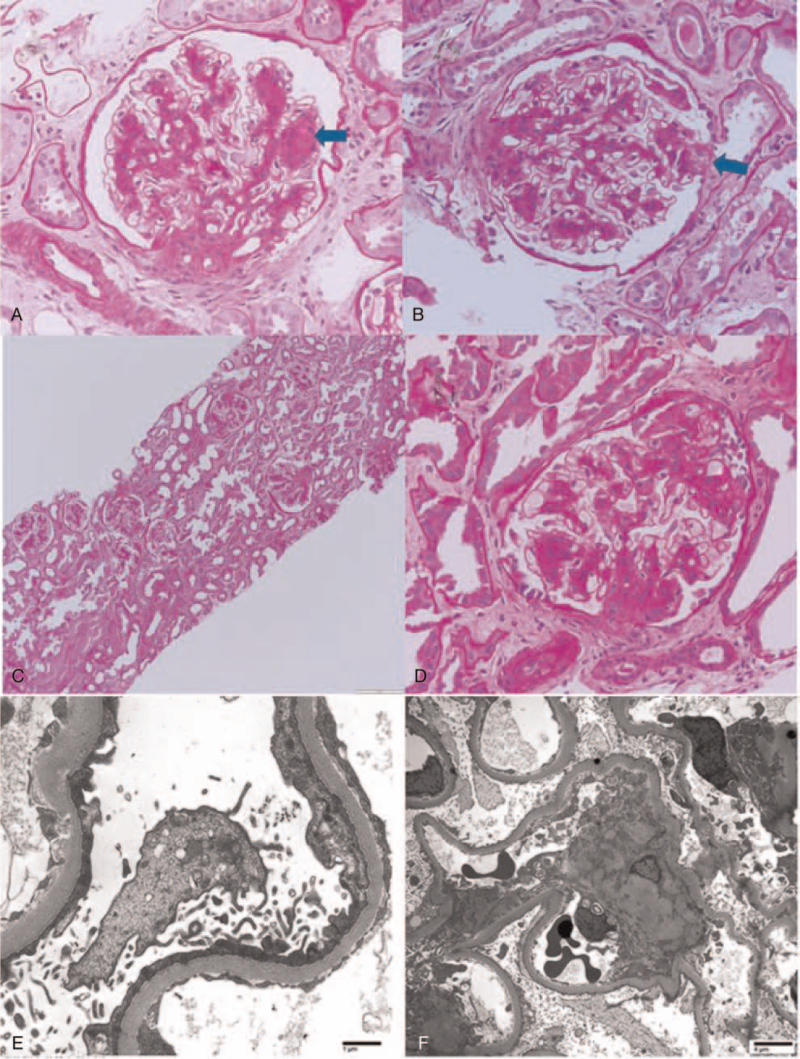
First and second renal biopsy findings by light microscopy and electron microscopy. A and B represent first biopsy (PAS stain) which show nodular glomerular mesangial sclerosis and relatively mild hypercellularity with Kimmelstiel Wilson nodule formation (arrow)(A), as well as segmental scar formation (B) with adhesion to the Bowman's capsule and associated foam cells (arrow). C and D represent second biopsy (PAS stain) which show similar histology findings to the first biopsy with nodular glomerulosclerosis (C) in most sampled glomeruli and focal segmental glomerulosclerosis (D). E and F are electron microscopic findings on the first biopsy which reveals over 90% of podocyte foot process effacement, no electron dense immune type deposits along the capillary loops or other places, and diffuse thickening of the glomerular basement membrane.

The renal biopsy contained 8 glomeruli, none of which were globally sclerotic. There was a variable increase in the mesangial matrix and cellularity in a nodular pattern (Fig. 1A). Two glomeruli showed segmental scars, with 1 demonstrating a tip lesion (Fig. 1B). There was no glomerulitis, endocapillary proliferation, thrombosis, necrosis, or crescent formation. The glomerular basement membranes were smooth with no spikes or double contours on PAS or Jones staining. Moderate interstitial fibrosis and tubular atrophy were also observed. The interstitium showed patchy mild inflammatory infiltrate predominantly in the medulla, composed of small numbers of lymphocytes, plasma cells, and occasional eosinophils. Focal tubular ectasia, epithelial attenuation, blebbing, cytoplasmic vacuolation, and rare mitosis were present A few proteinaceous casts and rare Tamm-Horsfall casts were observed. The arteries showed mild intimal fibrosis. Arteriolar hyalinosis was also observed. Immunofluorescence showed no granular immune staining in the glomeruli; however, diffuse nuclear staining by polytypic IgG was observed in the tubular epithelial cells, compatible with the patient's high ANA titers. Ultrastructural analysis (Fig. 1E and 1F) revealed diffuse thickening of glomerular basement membranes, ranging from 437 nm to 1187 nm. (753 ± 195 nm, based on 26 random measurements). No electron-dense immune type deposits were present in the mesangium, subepithelial, or subendothelial regions along the capillary loops. The podocytes showed microvillous transformation and diffuse foot process effacement of over 90% of the capillary loop surfaces. No electron-dense immune-type materials were observed in the interstitium or along the tubular basement membranes. The overall findings documented diabetic glomerulopathy and focal segmental glomerulosclerosis. Although the patient had autoimmune disease symptoms and a history of positive ANA and anti-dsDNA, there was no evidence of an immune complex deposition-mediated process in the glomerular compartment or in other renal spaces.

### 2.3 Initial treatment

The patient's nephrotic proteinuria was attributed to diabetic nephropathy with superimposed focal segmental glomerulosclerosis of uncertain etiology. The patient was started on ACEI and diuretics. Steroid therapy (15 mg/day) was discontinued. The patient was discharged to home with close follow-up with her nephrologist.

### 2.4 Subsequent presentation

A number of clinical laboratory test results were available 2 weeks after the biopsy. Positive serology results included anti-dsDNA antibody at 103 IU/ml (ref <29); anti-histone antibody at 8.5 U (ref <1); anti-Smith antibody at 1.8 AI (ref <0.9); SSA antibody at 2 AI (ref <=0.9); anti-centromere antibody at 1.8 AI (ref <1); and RNP antibody at > 8 AI (ref <=0.9). Other autoantibodies, including Scl70, SSB, Jo-1, and anti-PLA2R, were negative. Results of HIV, hepatitis A, B, and C panels were obtained at a later time and were all negative.

One month after the first biopsy, the patient presented to the emergency department due to blurry vision, headache, and elevated blood pressure. The patient reported that her blood pressure was in the 150's systolic over the past few days, and that she had bilateral headache (8/10) and sudden onset of blurred vision in her left eye. Ophthalmology was consulted, and the eye findings were diagnosed as hypertensive retinopathy. Laboratory tests showed severe electrolyte derangements including an increase in serum creatinine (3.3 mg/dL) with hyperkalemia (6.2 mmol/L) and hypobicarbonatemia (19 mEq/L). After admission, despite electrolyte corrections and discontinuation of ACEI, her serum creatinine increased further to 5.3 mg/dL, with her eGFR further reduced from 15 mL/min/1.73 m^2^ at presentation to 9 mL/min/1.73 m^2^. Her nephrotic proteinuria remained massive, with albumin down to 1.0 g/L and urine protein/creatinine ratio of 14.1 g/g Cr and albumen/creatinine ratio of 8224 mg/g Cr. Hemoglobin was 6.7 g/dL; hematocrit, 19.5%; platelet count, 87 × 10e9/L (Table 1); WBC, 4.2 x10e9/L (Table 1); and a Coombs test result was positive. As mentioned above, the serology tests ordered at the time of biopsy had been returned as positive for anti-dsDNA, anti-histone, anti-Smith, and anti-RNP antibodies. The clinical likelihood of SLE was considered. Due to the continued worsening of renal function, another renal biopsy was performed, which showed similar histological findings (Fig. 1C and 1D) to that of the first biopsy, including nodular glomerular sclerosis, focal segmental glomerulosclerosis, and no significant immune type staining. Integrating the full record of the patient's clinical presentation, all the test results, and the non-responsiveness to the treatment so far, the histopathology findings were considered most compatible with lupus podocytopathy with an FSGS pattern, superimposed on a pre-existing diabetic glomerulopathy.

**Table 1:** Laboratory values before and after diagnosis of lupus podocytopathy.

	Initial presentation	One month after ACEI treatment	One month after LP diagnosis and steroid treatment	Reference range
White blood cell count (x10^9^/L)	11.6	4.2	12.4	4–10.5
Hemoglobin (g/dL)	9.3	6.7	9.2	12–16
Platelet count (10^9^/L)	150	87	392	150–450
Serum albumin (g/dL)	1.8	1.0	2.8	3.5–5.0
Urine albumin/creatinine (g/g)	4.3	8.2	3.0	0–0.03
Serum creatinine (mg/dL)	1.9	3.3–5.3	1.3	0.4–1.4
eGFR^∗^ (ml/min/1.73 m^2^)	32.5	15	>60	>60

eGFR = estimated Glomerular Filtration Rate.

### 2.5 Adjusted treatment and outcome

**Figure 2:**
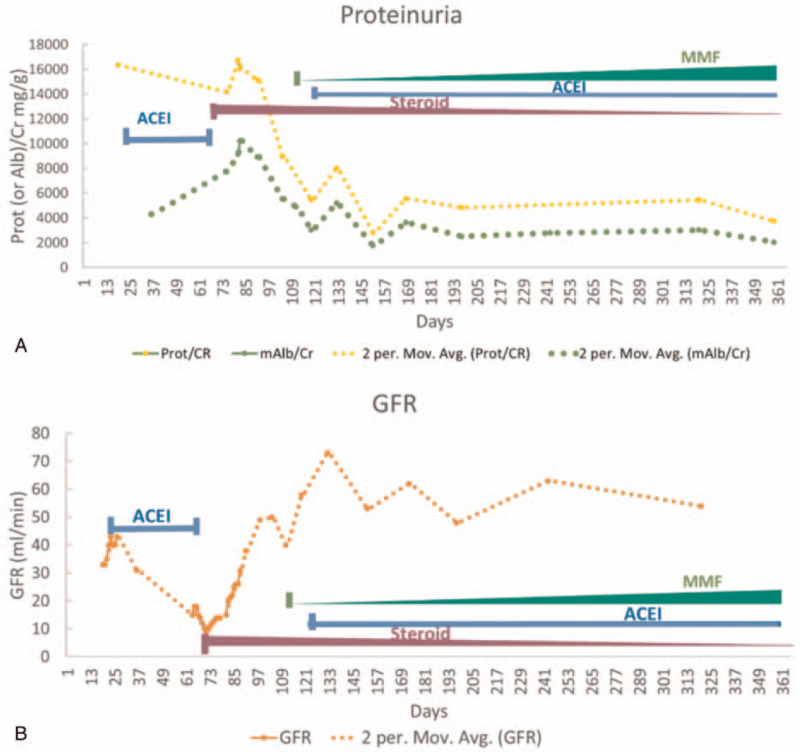
Treatment timelines and treatment effects on proteinuria and renal function. ACEI (Lisinopril) was used as monotherapy after the 1st biopsy (around day 22) and was ineffective for proteinuria control (A) or renal function preservation (B). After the 2nd renal biopsy (around day 67), high dose steroid (prednisolone 60 mg/day) was initiated followed by subsequently significant reduction of proteinuria (A) and improvement of GFR (B). One month later, MMF was added by gradually increased dosage (500 mg bid for 2 weeks, 750 mg bid for 1 month, and 1000 mg bid afterwards) while steroid was tapering (from 60 mg/day gradually reduced to 10 mg/day during a period of 295 days and remained on that dosage till present), ACEI was also added soon after MMF at a dosage of 10 mg per day. The renal function (GFR) (B) and proteinuria (A) have been stable for the afterwards over 10 month follow up.

The patient was started on high-dose prednisone 60 mg/dL per day. Her creatinine level started to improve, and the patient was discharged with continued daily high-dose prednisone treatment. One month later, her renal function, anemia and clinical symptoms were all significantly improved, with serum creatinine of 1.3 mg/dL, eGFR above 60 mL/min/1.73 m^2^, hemoglobin 9.2 g/dL, platelet count 392 x 10e9/L, and WBC 12.4 × 10e9/L (Table 1). Her urine albumin/creatinine ratio was 3015 mg/g Cr (Table 1) and urine protein/creatinine ratio was 5.4 g/g. ACEI was restarted (10 mg/day) along with mycophenolate mofetil (starting at 1 g/day and increased to 2 g/day) to permit tapering of corticosteroids. The treatment timelines and effects are illustrated in Figure 2, with proteinuria and GFR as readouts. Ten months after the adjusted therapeutic regimen, renal function remained stable and proteinuria was further reduced with a urine albumin/creatinine ratio of 2055 mg/g Cr (Fig. 2A) and a urine protein/creatinine ratio of 3.7 g/g Cr, while her GFR was maintained in the range of 50 to 60 mL/min/1.73 m^2^ (Fig. 2B). The persistent residual proteinuria may be due to the underlying diabetic nephropathy, or it may be due to a combination of diabetic glomerulosclerosis and the fixed FSGS lesions of LP.

## 3 Discussion

In this report, we describe the case of a 24 year old woman who developed the sudden onset of nephrotic syndrome, which was initially diagnosed on renal biopsy as diabetic nephropathy with superimposed FSGS, but was later refined with additional information for a diagnosis of lupus podocytopathy superimposed on diabetic glomerulosclerosis. This case raises difficult diagnostic and treatment issues. This patient had a long history of poorly controlled type 1 diabetes as well as a disease condition characterized by skin rash and polyarthritis with a positive ANA, which was thought to be psoriatic in nature. She had no prior history of lower extremity edema until ankle swelling developed suddenly after a switch from adalimumab to low-dose prednisone for her arthritis/skin rash. There were several initial clinical differentials for her nephrosis, including diabetic nephropathy and possible lupus nephritis due to the coexisting evidence of connective tissue disease. In addition, her recent therapy with Adalimumab also raised concern for possible drug-induced lupus-like nephritis, based on prior case reports that associated the anti-TNF therapies with the development of proliferative lupus nephritis during treatment for Rheumatoid Arthritis.^[3]^ In the current case, in contrast to those cases of Adalimumab drug-induced lupus-like nephritis reported in the literature, the worsening of nephrosis occurred after discontinuation of the drug. While the initial biopsy did indeed reveal changes consistent with diabetic glomerulosclerosis, there was also superimposed FSGS. There was no evidence of inflammatory or immunofluorescent changes featured in lupus nephritis or lupus-like nephritis. It was difficult to determine whether FSGS represented a primary process or a superimposed lesion secondary to advanced diabetic nephropathy. The initial treatment plan was to start high-dose steroids, presuming that FSGS was of primary etiology. The plan was rejected by the patient because of her uneasiness that prednisone, which started after adalimumab discontinuation, was potentially the cause of her swelling. There was also concern about the adverse effects of high-dose steroids on her already poorly controlled diabetic condition. At that time, it was decided to tentatively treat her diabetic nephropathy mainly, with intensified insulin therapy, ACEI, and diuretics, with close monitoring of her disease progression. The subsequent worsening of renal function, accelerated hypertension, persistent nephrosis, and newly developed evidence of autoimmune hemolytic anemia within 1 month of treatment, however, indicated ineffective treatment. Due to the continued worsening of serum creatinine, a second renal biopsy was performed to evaluate for interval changes and it showed histopathological findings similar to those of the first biopsy. Given this clinical presentation and her updated serology results with positive autoantibodies against dsDNA, histone, and Smith antigens, a diagnosis of SLE with lupus podocytopathy superimposed on diabetic nephropathy was made after re-evaluating the renal biopsy findings. Therapy with high-dose prednisone was initiated, followed by the gradual addition of mycophenolate mofetil. The subsequent course showed a rapid improvement in renal function (Creatinine from 5.3 mg/dL to 1.3 mg/dL) and a marked decrease in proteinuria (albumin/creatinine ratio from 8.2 g/g Cr to 2.05 g/g Cr). The cause of residual proteinuria (albumin/creatinine, 2–3 g/g), however, remains unclear. The possibility of persistent baseline proteinuria secondary to the well-established diabetic nephropathy versus a less than optimal therapeutic response to steroid therapy for lupus podocytopathy of the FSGS form were both considered. Therefore, it is difficult to determine whether the patient's LP was in partial or total remission.

The term LP was coined in the early 2000's to describe the phenomenon of nephrotic syndrome in patients with SLE characterized by diffuse glomerular podocyte foot process effacement without immune deposits in the capillary walls.^[4]^ LP was previously considered as the coincidence of minimal change disease or focal segmental glomerulosclerosis superimposed on lupus nephritis.^[5–9]^ However, several case series studies in the early 2000's found that the frequency of these cases was much higher than that expected for 2 diseases being coexistent and that the onset of nephrotic syndrome frequently correlated with the onset of clinical activities of SLE,^[4,10,11]^ prompting the proposal that LP was more likely a manifestation of active SLE than just the coincidence of 2 diseases. In the largest cohort study with 50 LP patients in 2016, the authors compared clinical-morphology features, treatment responses and outcomes between groups with glomerular minimal change/mesangial proliferation and FSGS, and showed that the FSGS form of LP is associated with higher incidence of AKI, more severe tubular-interstitial lesions and much lower remission rate.^[12]^ Subsequent case reports on LP revealed variable presentations of LP with concurrent extra renal or serologic activities, before or after clinical SLE diagnosis, casting challenges on the LP diagnosis. For example, LP has been reported as a co-current presentation with chylous ascites as the manifestation of SLE in a 3 year old girl,^[13]^ podocytopathy presenting as TTP-like syndrome in a 45 year old woman with subsequently diagnosed SLE,^[14]^ or collapsing podocytopathy presenting with heamophagocytic lymphohisocytosis as the manifestation of SLE.^[15]^ In addition, hydroxychloroquine has also been implicated to induce podocytopathy in SLE patients on maintenance therapy.^[16]^ LP has been reported to transform to or from other forms of lupus nephritis, sometimes years apart, most often with membranous lupus nephritis,^[17,18]^ but can also occur with diffuse mesangial proliferative lupus nephritis.^[19]^

In the treatment of LP, high-dose glucocorticoids (GC) with or without other immunosuppressive drugs are considered to be the first-line therapeutic agents. A large case series study with 50 LP patients from 2015^[20]^ showed that the remission of LP could be induced by GC monotherapy or GC plus other immunosuppressive agents, although the maintenance of remission was achieved more frequently with combination regimens. In other studies, calcineurin inhibitors (cyclosporine A and tacrolimus) have been shown to be beneficial in reducing proteinuria^[21]^ and to achieve a significantly higher complete remission rate and better long-term renal outcome in patients with extensive foot process effacement^[22]^ Rituximab has been proposed as a second-line agent to be used for reducing relapse rate and corticosteroid exposure, based on limited data.^[23]^ In a case report of a pediatric patient with steroid-resistant nephrotic syndrome, treatment with cyclosporine and an angiotensin receptor blocker was described to be effective for nephrotic proteinuria.^[24]^ In another LP case in an adult female, double filtration plasmapheresis in addition to oral steroid was reported to accelerate the patient's recovery from nephrotic syndrome and acute renal failure.^[25]^

Treatment options for nephrotic syndrome may vary based on the different pathogenesis of podocytopathies in variable disease conditions such as minimal change disease (MCD), primary focal segmental glomerulosclerosis, membranous glomerulopathy, diabetic glomerulopathy, amyloidosis, or lupus nephritis, among others. The pathogenesis of LP is still not fully understood. It is speculated that factors causing podocyte damage in LP are similar to those contributing to MCD or primary FSGS,^[1]^ which may include but may not be limited to aberrant T cell functions with alternatingly produced cytokines and lymphokines as proposed in MCD,^[26]^ or some circulatory factors^[27–29]^ such as soluble urokinase plasminogen activating receptor (suPAR),^[30]^ as proposed in primary FSGS. Interferon-alpha and several other proinflammatory cytokines are also suspected to play important roles in the pathogenesis of LP.^[31,32]^ In contrast, the pathogenesis of podocytopathy in diabetic glomerulopathy is considered to be due to factors such as hyperglycemia, advanced glycosylated end products, angiotensin II, and aldosterone, which may cause oxidant injury ^[33–36]^ leading to increased expression of TGF-beta and reduced production of VEGF,^[37–39]^ resulting in low podocyte density and podocyte dysfunction.^[40–45]^ Therefore, treatment for LP is similar to that for MCD or primary FSGS with glucocorticoid alone or combined with another immunosuppressive agent as first-line therapy, whereas treatment for nephrotic proteinuria in diabetic nephropathy is managed by glycemic control and ACEI. In our case presented here, treatment failure by ACEI and subsequent success in proteinuria reduction by a large dose of steroid further supports the diagnosis of LP.

## 4 Conclusion

Compared to previous case reports of lupus podocytopathy, which have been histologically described as minimal change, mesangial proliferative or focal segmental glomerulosclerosis patterns, here we present a case that shows that lupus podocytopathy may coexist with other non-lupus glomerular diseases that demonstrate more complex glomerular morphologies with associated nephrotic syndrome, which could lead to missed LP diagnosis. To our knowledge, this is the first case report of lupus podocytopathy diagnosed in the background of fully developed diabetic glomerulonephropathy. It is important that pathologists and clinicians are aware of such an occurrence, as they could have critical diagnostic and treatment significance.

## Author contributions

**Conceptualization:** Lin Liu, John E. Tomszewski.

**Data curation:** Lin Liu, Brian Murray.

**Investigation:** Lin Liu, Brian Murray.

**Supervision:** John E. Tomszewski.

**Writing – original draft:** Lin Liu.

**Writing – review & editing:** Brian Murray, John E. Tomszewski.
